# A nomogram to predict mortality in patients with severe fever with thrombocytopenia syndrome at the early stage—A multicenter study in China

**DOI:** 10.1371/journal.pntd.0007829

**Published:** 2019-11-25

**Authors:** Lin Wang, Gang Wan, Yi Shen, Zhenghua Zhao, Ling Lin, Wei Zhang, Rui Song, Di Tian, Jing Wen, Yongxiang Zhao, Xiaoli Yu, Li Liu, Yang Feng, Yuanni Liu, Chunqian Qiang, Jianping Duan, Yanli Ma, Ying Liu, Yanan Liu, Chong Chen, Ziruo Ge, Xingwang Li, Zhihai Chen, Tianli Fan, Wei Li

**Affiliations:** 1 Center of Infectious Disease, Beijing Ditan Hospital, Capital Medical University, Beijing, China; 2 Statistics Room, Beijing Ditan Hospital, Capital Medical University, Beijing, China; 3 Department of Infectious Diseases, Dandong Infectious Disease Hospital, Dandong, China; 4 Department of Infectious Disease, Taian City Central Hospital, Taian, China; 5 Department of Infectious Disease, Yantai City Hospital for Infectious Disease, Yantai, China; 6 Department of Infectious Disease, Qing Dao No. 6 People’s Hospital, Qingdao, China; 7 Clinical Laboratory, Beijing Ditan Hospital, Capital Medical University, Beijing, China; 8 Graduate School of Capital Medical University, Beijing, China; 9 Interventional Therapy Oncology, Beijing Ditan Hospital, Capital Medical University, Beijing, China; University of Liverpool, UNITED KINGDOM

## Abstract

**Background:**

Severe fever with thrombocytopenia syndrome (SFTS) caused by the SFTS virus is an emerging infectious disease that was first identified in the rural areas of China in 2011. Severe cases often result in death due to multiple organ failure. To date, there are still numerous problems remain unresolved in SFTS, including unclear pathogenesis, lack of specific treatment, and no effective vaccines available.

**Aim:**

To analyze the clinical information of patients with early-stage SFTS and to establish a nomogram for the mortality risk.

**Methods:**

Between April 2011 and December 2018, data on consecutive patients who were diagnosed with SFTS were prospectively collected from five medical centers distributed in central and northeastern China. Multivariable Cox analyses were used to identify the factors independently associated with mortality. A nomogram for mortality was established using those factors.

**Results:**

During the study period, 429 consecutive patients were diagnosed with SFTS at the early stage of the disease (within 7 days of fever), among whom 69 (16.1%) died within 28 days. The multivariable Cox proportional hazard regression analysis showed that low lymphocyte percentage, early-stage encephalopathy, and elevated concentration of serum LDH and BUN were independent risk factors for fatal outcomes. Received-operating characteristic curves for 7-, 14-, and 28-days survival had AUCs of 0.944 (95% CI: 0.920–0.968), 0.924 (95% CI: 0.896–0.953), and 0.924 (95% CI: 0.895–0.952), respectively. Among low-risk patients, 6 patients died (2.2%). Among moderate-risk patients, 25 patients died (24.0%, hazard ratio (HR) = 11.957). Among high-risk patients, the mortality rate was 69.1% (HR = 57.768).

**Conclusion:**

We established a simple and practical clinical scoring system, through which we can identify critically ill patients and provide intensive medical intervention for patients as soon as possible to reduce mortality.

## Author summary

We established a SFTS nomogram scoring system, which is the first nomogram for this disease. According to this nomogram, patients were divided into three levels of mortality risk: low, moderate, and high. This scoring system is helpful to identify critically ill patients, allowing for early intervention and intensive care, which may contribute to reducing the mortality of SFTS.

## Introduction

Severe fever with thrombocytopenia syndrome (SFTS) caused by the SFTS virus (SFTSV) is an emerging infectious disease that was first identified in the rural areas of China in 2011 [1]. The patients are all from the hilly areas of central and northeastern China, and are mostly farmers working in the endemic areas. Most of the cases occur between April and October and Haemaphysalis longicornis is considered the most likely transmission vector [2–4]. There were also reports of clustered cases and outbreaks of nosocomial infections [5,6], indicating that except for the tick bite, there is a potential risk of human-to-human transmission [7–9]. The major clinical manifestations of SFTS are fever, fatigue, thrombocytopenia and leucopenia, usually accompanied with myocardium impairment and elevated liver enzymes. Severe cases often die from multi-organ failure and the mortality rate of SFTS is 11.2%-30% [1,10–12].

The disease is fairly broadly distributed in China, is tick-borne, and is epidemics in more than 20 provinces [7,8] and with increasing numbers of cases each year [13]. Since the identification of the SFTSV in 2011, all patients are mandatorily reported by doctors within 24 h of diagnosis to the China Information System for Diseases Control and Prevention (CISDCP). In addition to its prevalence in rural area of central and northeastern China, this disease is widely distributed in South Korea [14] and Japan [15], two neighboring countries of China. In addition, other newly emerged phleboviruses were isolated from patients in USA in 2012, including the Heartland virus, a new phlebovirus, which is genetically similar to SFTSV [16]. In 2011–2016, a total of 5360 laboratory-confirmed patients were reported to the CISDCP. Annual patient numbers increased yearly and the numbers of affected counties increased sharply from 98 to 167 from 2011 to 2016 [17]. The scope of the disease is expanding, with China being the hardest hit country and the mortality rate remains high.

The management of SFTS still faces issues such as unclear pathogenesis, lack of specific treatment, and no approved vaccines. The clinical symptoms of SFTS are non-specific and often need to be differentiated from human anaplasmosis and hemorrhagic fever with renal syndrome (HFRS) caused by hantavirus, since those two diseases are also found in the same geographical areas [1]. Furthermore, laboratory diagnosis needs to be confirmed by the Centers for Disease Control and Prevention (CDC). In addition, there is still no consensus on the staging and classification of SFTS and, so far, there is no specific antiviral therapy. Therefore, identifying risk factors for death of SFTS can be helpful for further understanding of the viral pathogenesis and revealing the cause of death. Early identification of critically ill patients is very important to carry out symptomatic treatments to reduce the mortality rate. Therefore, there is an urgent need for establishing a death risk predictive scoring system, assessing the severity of illness, and predicting the risk of death in order to take effective public health measures to control the epidemic and to recognize which patients require early interventions. Public health measures could eventually include better education for better recognition of the disease by front-line physicians.

In the present prospective cohort study, we analyzed the clinical information of patients with SFTS at the early stage of the disease. We compared the data between the survival and mortality groups using univariable and multivariable Cox regression analyses in order to screen for significant critical early-stage factors related to death. Based on this analysis, we established a SFTS nomogram scoring system, which is intuitional, fast, practical, and operable [18,19], and is the first nomogram for this disease. Using this scoring system, we divided the patients into three levels of mortality risk: low, moderate, and high. This nomogram can identify critically ill patients at the early stage and could allow mortality reduction.

## Patients and methods

### Patients

Between April 2011 and December 2018, data on consecutive patients who presented with acute fever (temperature >37.5°C for over 24 h) and thrombocytopenia (Platelet count <100×10^9^ /L) were prospectively collected from the five medical centers distributed in central and northeastern China: Beijing Ditan Hospital, Dandong Infectious Disease Hospital, Taian City Central Hospital, Qing Dao No. 6 People’s Hospital, and Yantai City Hospital for Infectious Disease. In total we enrolled 967 patients with clinical manifestation of fever accompanied by thrombocytopenia. Among them, 695 were patients diagnosed with SFTS, among whom 429 patients visited the hospitals at the early-stage (within 7 days from disease onset), which were the subjects of this study. This study was approved by the local Ethics Committee of the lead center, Beijing Ditan Hospital, Capital Medical University (number 2014–003) and complied with the principles of the Declaration of Helsinki. Informed consent was obtained from all patients or their immediate relatives for sample and data collection and their use for research.

Patients diagnosed with SFTS were included in the study. The diagnosis of SFTS was confirmed by: 1) acute fever (temperature >37.5 °C for over 24 h) with thrombocytopenia (platelet count <100×10^9^ /L); and 2) laboratory-confirmed SFTSV infection by detection of viral RNA (fluorescent probe PCR, BGI-GBI, Beijing, China), and/or virus-specific IgM antibody in the peripheral blood (ELISA, Zhongshan Bio-Tech CO., LTD, Guangdong, China) [20]. Those two tests are routinely used for the diagnosis of SFTS in China. Routine validation is carried out using samples sent by the China Information System for Diseases Control and Prevention. The exclusion criteria were: 1) laboratory-confirmed other pathogen infection including Platts and Mori rickettsia, Orientia tsutsugamushi, Hantavirus, etc.; or 2) history of acute or chronic blood system diseases.

### Treatment

All patients were given systemic support treatment, including infusion of blood products, granulocyte colony-stimulating factor (G-CSF), and Chinese traditional medicine. For patients with severe SFTS, glucocorticoid and/or hemodialysis were performed. All the patients received supportive measures, including rest, nutritional support, and electrolyte balance maintenance.

### Data collection and definitions

Demographic and clinicopathological data were collected. Blood samples were collected on admission and throughout the disease course. For clinical data collection, we designed a medical record form and database in Epidata (Centers for Disease Control, Atlanta, GA, USA) that contains information of epidemiologic, clinical manifestations, physical examinations, and laboratory parameters. Demographic factors and date of onset were included. All data were entered by trained study staffs. Patients who discontinued therapy or were discharged from hospital because of personal reasons were followed till 28 days from disease onset time. The primary outcome was 28 days all-caused death.

The date of disease onset was defined as the day fever was noticed (self-reported). Early stage SFTS was defined as within 7 days from disease onset. Encephalopathy was defined as an altered mental status that persisted for more than 24 h, including lethargy, irritability, or a change in personality and behavior [21]. Severe SFTS was defined as SFTS combined with multiple organ failure. Death was defined as death from any cause.

### Statistical analysis

All statistical analyses were conducted with SPSS 19.0 (IBM, Armonk, NY, USA). Continuous variables are presented as mean ± standard deviation or medians with interquartile ranges, while categorical variables are presented as frequencies or percentages of events. The two-sample t test or Mann-Whitney U test was used to determine the relationship between the survival and mortality for continuous data. The Pearson chi-square and Fisher’s exact tests were used to compare differences in proportion between groups, as appropriate. Independent risk factors were identified by univariable and multivariable Cox regression analyses for predicting mortality.

A nomogram was formulated based on the results of the multivariable Cox regression analyses. The regression coefficient of different variables in the Cox regression equation and the extreme difference of the variables were used to calculate the product of the regression coefficient and the range of the different variables. Assuming that the largest product variable is 10 points, and assuming that the variable is a reference variable, the score corresponding to each unit of the variable could be calculated according to the range. The other variables were multiplied by the score corresponding to each unit of the reference variable according to the ratio of the regression coefficient to the regression coefficient of the reference variable, and the score corresponding to each unit of the variable was calculated. After calculating the score for each variable, the nomogram was formulated using the nomogram function of the RMS packages [22] in R version 3.0.2 (The R Project for Statistical Computing, www.r-project.org).

The performance of the nomogram was evaluated by the concordance index (C-index). The maximum C-index value is 1.0, which indicates a perfect prediction model, whereas 0.5 indicates a random chance to correctly predict outcome by the model. The model was validated using bootstrapped resampling to quantify any overfitting. The calibration curve for the nomogram used 1000 repeated samples to obtain the corresponding actual incidence rate and 95% confidence interval (CI) at different probability levels to evaluate the consistency of model prediction. Bootstrap sampling was performed using the RMS packages in R version 3.0.2 (The R Project for Statistical Computing, www.r-project.org). Calibration curves of the nomogram for 7-, 14-, and 28-days were applied to assess the agreement between predicted and observed survival. Clinical survival outcomes were assessed by the Kaplan-Meier analysis and prognostic groups were compared by the log-rank test. The X-tile software package [23] was used to search the cut-off value of nomogram points for grading death risk. All statistical tests were two-sided with a statistical significance level set at p values of < 0.05.

## Results

### Demographic and clinical characteristics of the patients with SFTS

During the study period, 429 consecutive patients were diagnosed with SFTS at the early stage of the disease (within 7 days of fever), among whom 69 died within 28 days. The mortality rate was 16.1%. The survival group included 201 women (46.9%) and 228 men (53.2%), while the mortality group included 26 women (37.7%) and 43 men (62.3%). There was no significant sex difference between the two groups. The mean age of all patients was 60.8±12.1 years. Notably, the mean age in patients who died was significantly higher than in those who survived (68.3 vs. 59.3; p < 0.05) (Table 1). Most of the cases occurred between May and October during the year. The patients were all from the hilly areas of central and northeast China, and were mostly farmers working in the endemic areas.

**Table 1:** Comparison of gender, age, clinical symptoms, and laboratory parameters in the survival and mortality groups.

	All (n = 429)	Survival (n = 360)	Fatal (n = 69)	P
**Sex, n (%)**				
Male	228 (53.2)	185 (51.4)	43 (62.3)	0.096
Female	201 (46.9)	175 (48.6)	26 (37.7)	
**Age (years)** ^§^	60.8±12.1	59.3±11.7	68.3±11.0	<0.001
**Clinical symptoms, n (%)**				
Chills	152 (35.4)	128 (35.6)	24 (34.8)	0.902
Headache ^§^	125 (29.1)	115 (31.9)	10 (14.5)	0.001
Dizziness	121 (28.2)	108 (30.0)	13 (18.8)	0.059
Myalgia	178 (41.5)	153 (42.5)	25 (36.2)	0.333
Anorexia	322 (75.1)	274 (76.1)	48 (69.6)	0.250
Nausea	200 (46.6)	174 (48.3)	26 (37.7)	0.104
Vomiting	70 (16.3)	57 (15.8)	13 (18.8)	0.536
Bloating	11 (2.6)	10 (2.8)	1 (1.5)	>0.99
Diarrhea	81 (18.9)	67 (18.6)	14 (20.3)	0.744
Cough	75 (17.5)	64 (17.8)	11 (15.9)	0.713
Sputum	49 (11.4)	42 (11.7)	7 (10.1)	0.716
Chest distress	33 (7.7)	30 (8.3)	3 (4.4)	0.255
Hypourocrinia	34 (7.9)	31 (8.6)	3 (4.3)	0.230
Encephalopathy ^§^	109 (25.4)	52 (14.4)	57 (82.6)	<0.001
Flush	83 (19.4)	71 (19.7)	12 (17.4)	0.653
Pharyngeal hyperemia	141 (32.9)	120 (33.3)	21 (30.4)	0.639
Petechia/Ecchymosis	37 (8.6)	28 (7.8)	9 (13.0)	0.153
Lymphadenopathy	101 (23.5)	83 (23.1)	18 (26.1)	0.587
Hepatosplenomegaly	27 (6.3)	24 (6.7)	3 (4.4)	0.596
Abdominal tenderness	53 (12.4)	45 (12.5)	8 (11.6)	0.834
**Laboratory parameters**				
WBC (10^9^/L)	2.2 (1.5,3.9)	2.3 (1.5,3.9)	1.9 (1.4,4.0)	0.342
NEU (%) *	61.03±18.34	60.06±18.35	65.95±17.64	0.016
NEU (10^9^ /L)	1.2 (0.8,2.4)	1.2 (0.8,2.4)	1.4 (1.0,2.7)	0.181
LYM (%) *	29.26±14.16	30.58±14.32	22.52±11.17	<0.001
LYM (10^9^/L)	0.6(0.4,1.0)	0.6(0.4,1.1)	0.5(0.3,0.7)	0.009
MON (%)	6.3(3.0,10.7)	6.6(3.4,10.9)	3.9(1.6,9.0)	0.001
MON (10^9^/L)	0.1(0.1,0.3)	0.1(0.1,0.3)	0.1(0.0,0.2)	0.012
RBC (10^12^/L) *	4.57±0.66	4.57±0.60	4.52±0.89	0.647
Hb (g/L) *	138.74±20.28	137.98±20.75	142.71±17.26	0.082
PLT (109 /L)	55.0(40.0,71.0)	56.0(42.0,73.0)	41.7(31.0,64.0)	0.002
LDH (U/L)	605.5 (384.0,900.0)	536.0 (360.0,864.0)	900.0 (639.0,1244.0)	<0.001
CK (U/L)	504.0 (184.0,1155.0)	455.0 (172.0,985.0)	1163.0 (247.0,2000.0)	<0.001
CK-MB (U/L)	23.9 (15.0,38.8)	22.6 (14.9,35.1)	35.0 (16.0,71.0)	0.001
K (mmol/L) *	3.79±0.55	3.74±0.52	4.09±0.61	<0.001
NA (mmol/L) *	133.27±5.53	133.35±5.39	132.78±6.30	0.477
Cl (mmol/L) *	97.80±5.19	97.78±4.98	97.89±6.32	0.902
BUN (mmol/L)	5.7 (4.1,8.0)	5.2 (3.9,7.3)	8.3 (6.1,13.1)	<0.001
sCr (μmol/L)	72.5 (59.0,95.0)	70.0 (58.0,90.0)	87.5 (65.0,142.7)	0.001
GLU (mmol/L) *	7.80±4.58	7.80±4.64	7.85±4.22	0.939
PT (s)	12.3 (11.2,13.5)	12.2 (11.2,13.3)	12.5 (11.7,14.3)	0.043
PTA (%) *	100.49±27.41	102.10±26.90	91.90±28.84	0.033
APTT (s) *	43.05±16.36	40.33±10.88	59.38±29.53	<0.001
ALT (U/L)	72.6 (40.0,133.0)	67.5 (38.0,122.5)	115.8 (67.0,261.0)	<0.001
AST (U/L)	146.0 (71.0,275.1)	125.2 (67.0,247.0)	416.0 (197.2,793.0)	<0.001
TBil (μmol/L)	10.1 (7.3,13.3)	9.8 (7.2,13.0)	12.7 (9.5,23.3)	<0.001
ALB (g/L) *	33.17±5.20	33.29±4.98	32.34±6.52	0.361

WBC white blood cell, NEU (%) neutrophil percentage, NEU neutrophil, LYM (%) lymphocyte percentage, LYM lymphocyte, MON (%) monocyte percentage, MON monocyte, RBC red blood cell, Hb hemoglobin, PLT platelet count, LDH lactate dehydrogenase, CK creatinine kinase, CK-MB creatinine kinase myocardial b fraction, K potassium, Na sodium, Cl chloride, BUN Blood urea nitrogen, sCr serum creatinine, GLU glucose, PT prothrombin time, PTA prothrombin activity, APTT activated partial thromboplastin time, ALT alanine aminotransferase, AST aspartate aminotransferase, TB total bilirubin, ALB albumin.

^§^ P<0.05

* t test was used to compare groups. Mean ± standard deviation.

### Manifestation during the early stage of the disease

Most of the patients visited the hospitals at the early stage of SFTS. They showed moderate fever and their average temperature at admission was 38.9±0.6°C. Body temperature at admission in the survival and mortality groups was comparable (38.9±0.6 vs. 38.9±0.5 °C, p > 0.05). Other major systemic symptoms included headache (29.1%), dizziness (28.2%), myalgia (41.5%) and chills (35.4%). Gastrointestinal symptoms included nausea (46.6%), vomiting (16.3%), diarrhea (18.9%), and bloating (2.6%). Some patients also had respiratory symptoms such as cough (17.5%), sputum (11.4%), and tachypnea (7.7%). Encephalopathy was identified in 109 (25.4%) patients, of which included 57 mortality cases. The incidence of encephalopathy in fatal cases was significantly higher than in those who survived (57/69 (82.6%) vs. 52/360 (14.4%), p < 0.05) (Table 1). On the other hand, the incidence of headache in the survival group was significantly higher than in the mortality group (31.9% vs. 14.5%, p < 0.05).

Abnormal physical examination findings at admission included pharyngeal hyperemia (32.9%), flushing (19.4%), abdominal tenderness (12.4%), lymphadenopathy (23.5%), hepatosplenomegaly (6.3%), and hemorrhages (8.6%). There were no significance differences of these findings between the survival and mortality groups.

### Comparison of laboratory parameters in surviving and fatal SFTS cases

All patients exhibited early leukopenia, thrombocytopenia, elevated liver enzymes, and impairment of myocardial functions. Patients with fatal outcomes had a higher percentage of neutrophils, and lower percentages and absolute counts of lymphocytes, monocytes, and platelets. As shown in Table 1, the levels of creatine kinase (CK), creatine kinase isozyme (CK-MB), lactate dehydrogenase (LDH), alanine aminotransferase (ALT), and aspartate aminotransferase (AST) were significantly increased and APTT was markedly longer in the mortality group compared with the survival group. Interestingly, unlike patients with HFRS, only a small number of patients with SFTS, especially in fatal cases, showed mild renal impairment. Levels of blood urea nitrogen (BUN) and serum creatinine (sCr) in fatal patients were significantly higher than in non-fatal cases (Table 1). Though the fatal group had a relatively higher concentration of serum potassium and bilirubin (TBil), and lower percentage of prothrombin activity, the levels were still within the normal range.

### Risk factors for fatal outcomes

Univariable Cox regression analyses revealed that older age, presence of encephalopathy, decreased percentage of lymphocytes, low PTA, elevated percentage of neutrophil, and elevated levels of CK, CK-MB, LDH (ULN), ALT, AST, BUN, sCr, and APTT were risk factors for fatal outcomes. The above variables were used for multivariable Cox regression analyses. The results indicated that low lymphocyte percentage, early-stage encephalopathy, and elevated concentration of serum LDH and BUN were independent risk factors for fatal outcomes. The detailed results of the multivariable analyses are shown in Table 2.

**Table 2:** Univariable and multivariable Cox regression analyses of features associated with fatal outcomes in patients with SFTS.

	Univariable analysis					Multivariable analysis				
	ß	P	HR	95%CI for HR		ß	P	HR	95%CI for HR	
				Lower	Upper				Lower	Upper
Sex	-0.408	0.101	0.665	0.409	1.082					
Age	0.071	<0.001	1.073	1.048	1.099	0.035	0.010	1.036	1.008	1.064
Headache	-0.279	0.314	0.757	0.440	1.302					
Encephalopathy	3.012	<0.001	20.330	11.091	37.265	2.567	<0.001	13.028	6.771	25.068
WBC (109/L)	-0.030	0.401	0.970	0.905	1.041					
NEU (%)	0.018	0.016	1.018	1.003	1.033					
NEU (109/L)	0.029	0.506	1.030	0.944	1.123					
LYM (%)	-0.043	<0.001	0.958	0.939	0.977	-0.038	<0.001	0.963	0.943	0.983
LYM (109/L)	-0.257	0.181	0.773	0.531	1.127					
MON (%)	-0.043	0.071	0.958	0.914	1.004					
MON (109/L)	-0.005	0.751	0.995	0.968	1.024					
RBC (1012/L)	-0.110	0.560	0.896	0.619	1.297					
Hb (g/L)	0.010	0.080	1.010	0.999	1.022					
PLT (109/L)	-0.001	0.800	0.999	0.990	1.008					
LDH (U/L)	0.001	<0.001	1.001	1.000	1.001					
CK (U/L)	0.000	0.001	1.000	1.000	1.000					
CK-MB (U/L)	0.004	0.001	1.004	1.002	1.006					
LDH (ULN)	0.554	<0.001	1.741	1.407	2.154	0.323	0.004	1.381	1.111	1.718
BUN (mmol/L)	0.074	<0.001	1.076	1.049	1.105	0.053	<0.001	1.055	1.026	1.084
sCr (μmol/L)	0.004	<0.001	1.004	1.002	1.006					
GLU (mmol/L)	-0.003	0.923	0.997	0.935	1.063					
PT (s)	-0.001	0.855	0.999	0.986	1.012					
PTA (%)	-0.013	0.023	0.987	0.976	0.998					
APTT (s)	0.031	<0.001	1.032	1.023	1.041					
ALT (U/L)	0.000	0.011	1.000	1.000	1.001					
AST (U/L)	0.001	<0.001	1.001	1.001	1.001					
ALB (g/L)	-0.033	0.266	0.968	0.913	1.026					

WBC white blood cell, NEU (%) neutrophil percentage, NEU neutrophil, LYM (%) lymphocyte percentage, LYM lymphocyte, MON (%) monocyte percentage, MON monocyte, RBC red blood cell, Hb hemoglobin, PLT platelet count, LDH lactate dehydrogenase, CK creatinine kinase, CK-MB creatinine kinase myocardial b fraction, LDH (ULN) Multiple of the normal upper limit of lactate dehydrogenase, BUN Blood urea nitrogen, sCr serum creatinine, GLU glucose, PT prothrombin time, PTA prothrombin activity, APTT activated partial thromboplastin time, ALT alanine aminotransferase, AST aspartate aminotransferase, ALB albumin.

### Prognostic nomogram for mortality risk

**Fig 1:**
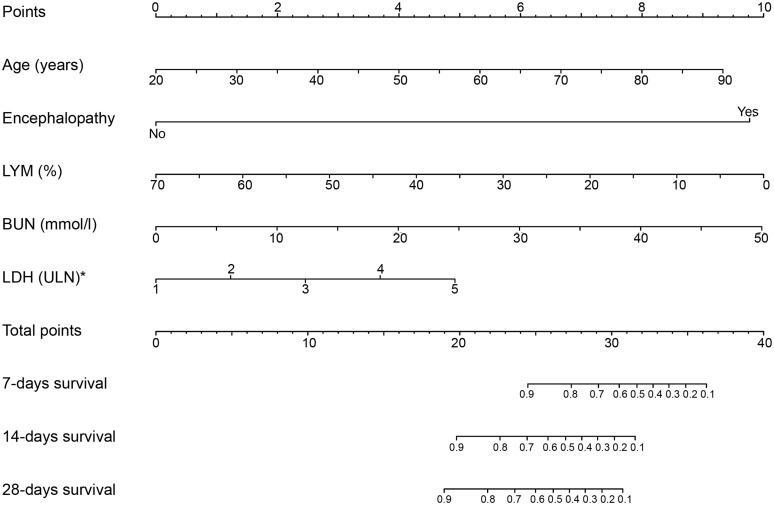
SFTS survival rate nomogram. To use the nomogram, the value of an individual patient is located on each variable axis, and a line is drawn upward to determine the number of points received for the value of each variable. The sum of these numbers is located on the total point axis, and a line is drawn downward to the survival axes to determine the probability of 7-, 14-, and 28-days survivals. LYM (%) lymphocyte percentage; LDH lactate dehydrogenase; BUN Blood urea nitrogen. *LDH (ULN) Multiple of the normal upper limit of lactate dehydrogenase.

The coefficients obtained from the Cox regression model were used to establish the nomograms for death (Fig 1). Each parameter within the variables was assigned a score. By adding up the total score from all the variables and locating it to the total point scale, we could determine the probabilities of death by drawing a vertical line to the total score. The nomograms included all the five risk factors screened by the multivariable Cox analysis: older age, low lymphocyte percentage, early-stage encephalopathy, and elevated serum LDH and BUN levels. Details concerning the point assignment for the nomogram and the prognostic score are shown in Table 3.

**Table 3:** Early-stage points of patients with SFTS and predicted survival rate.

Age	Points	Encephalopathy	Points	LYM (%)	Points	BUN (mmol/L)	Points	LDH (ULN)*	Points	Survival rate					
										Total points	7 days	Total points	14 days	Total points	28 days
20	0	No	0	70	0	0	0	1	0	36	0.1	32	0.1	31	0.1
30	1	Yes	10	60	1	10	2	2	1	35	0.2	30	0.2	29	0.2
40	2			50	3	20	4	3	2	34	0.3	29	0.3	28	0.3
50	4			40	4	30	6	4	4	33	0.4	28	0.4	27	0.4
60	5			30	6	40	8	5	5	32	0.5	27	0.5	26	0.5
70	6			20	7	50	10			30	0.6	26	0.6	25	0.6
80	8			10	9					29	0.7	24	0.7	24	0.7
90	9			0	10					27	0.8	23	0.8	22	0.8
										24	0.9	20	0.9	19	0.9

LYM (%) lymphocyte percentage; BUN Blood urea nitrogen; LDH lactate dehydrogenase.

* LDH (ULN) Multiple of the normal upper limit of lactate dehydrogenase

### Validation of the prognostic nomogram

**Fig 2:**
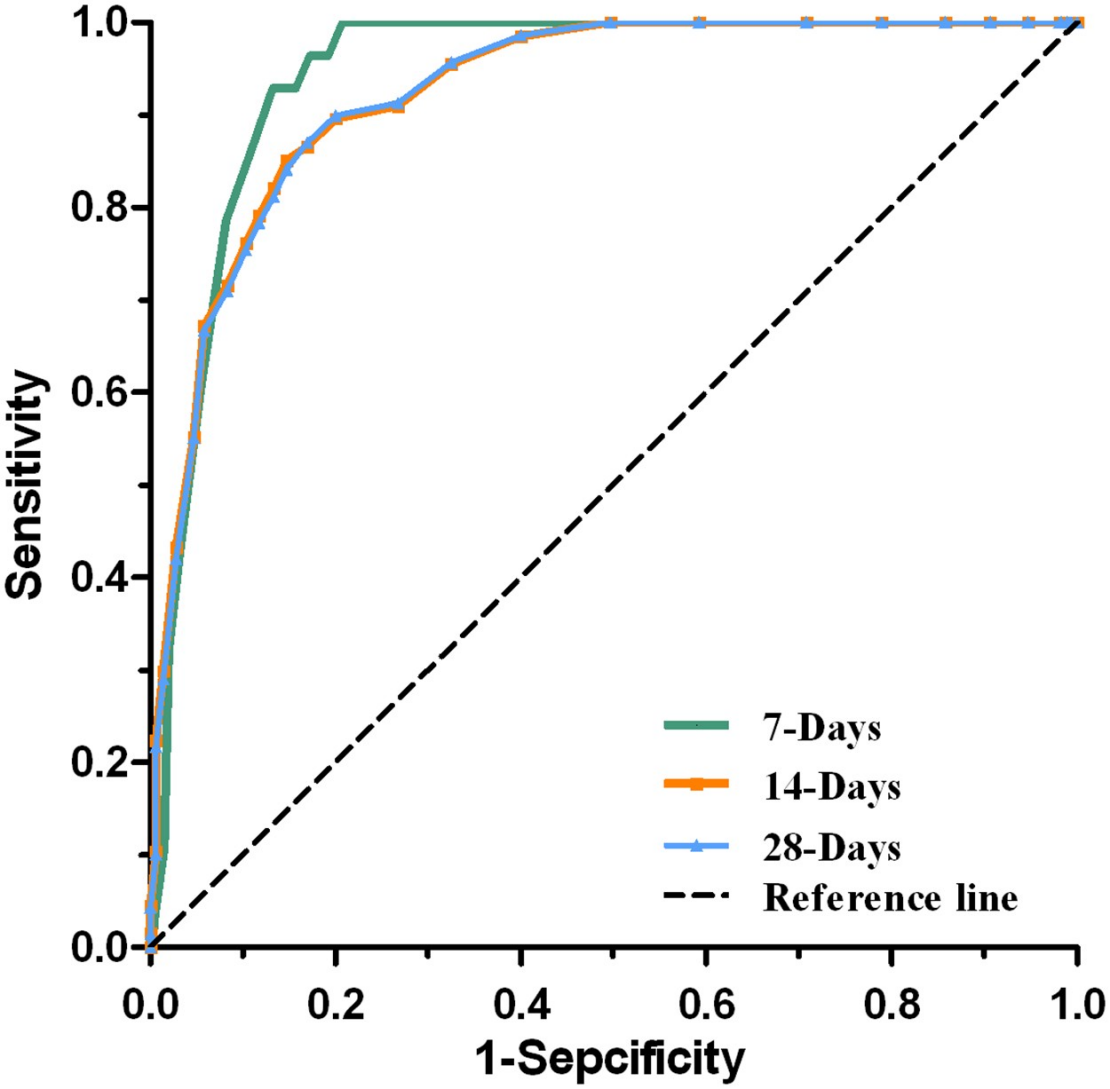
Predictive accuracy of the nomogram model. Receiver operating characteristics (ROC) curves of the multivariable logistic regression model for predicting 7-, 14-, and 28-days survivals in patients with SFTS.

**Fig 3:**
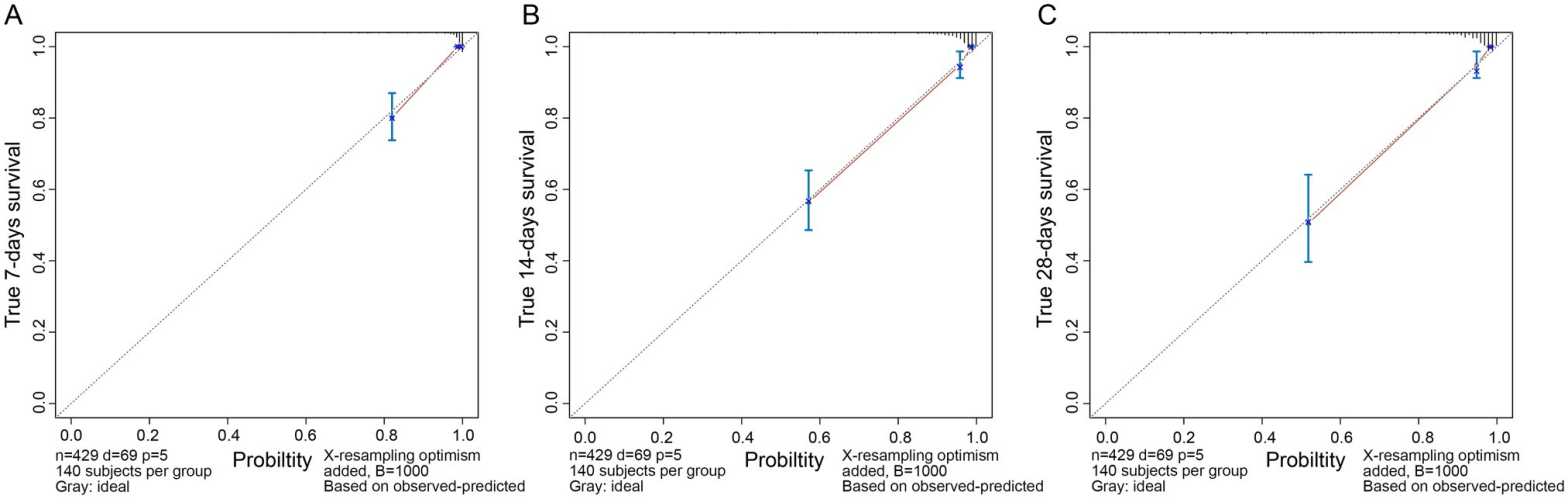
Calibration curves of overall survival at 7, 14, and 28 days for the primary cohort. Nomogram-predicted probability of survival is plotted on the x-axis, and the actual survival is plotted on the y-axis. Dashed lines along the 45° line through the point of origin represent the perfect calibration models where the predicted probabilities are identical to the actual probabilities. A. Overall survival at 7 days. B. Overall survival at 14 days. C. Overall survival at 28 days.

The C-index for the established nomogram to predict the death risk was 0.91 (95% CI: 0.87–0.93). ROC curves and calibration plots were performed to validate the predictive accuracy of the nomogram. The 7-, 14-, and 28-days survival ROC results had AUCs of 0.944 (95% CI: 0.920–0.968), 0.924 (95% CI: 0.896–0.953), and 0.924 (95% CI: 0.895–0.952), respectively (Figs 2 and 3 and Table 4), suggesting a good concordance and a reliable ability.

**Table 4:** AUC of the ROC curves of the nomogram model.

	AUC	95%CI for AUC		Goodness of Fit	
		Lower	Upper	LR	R^2^
7-Days	0.944	0.920	0.968	116.12	0.49
14-Days	0.924	0.896	0.953	208.79	0.54
28-Days	0.924	0.895	0.952	213.94	0.54

AUC: area under the receiver operating characteristics curve.

### Prognosis classification of patients with SFTS

**Fig 4:**
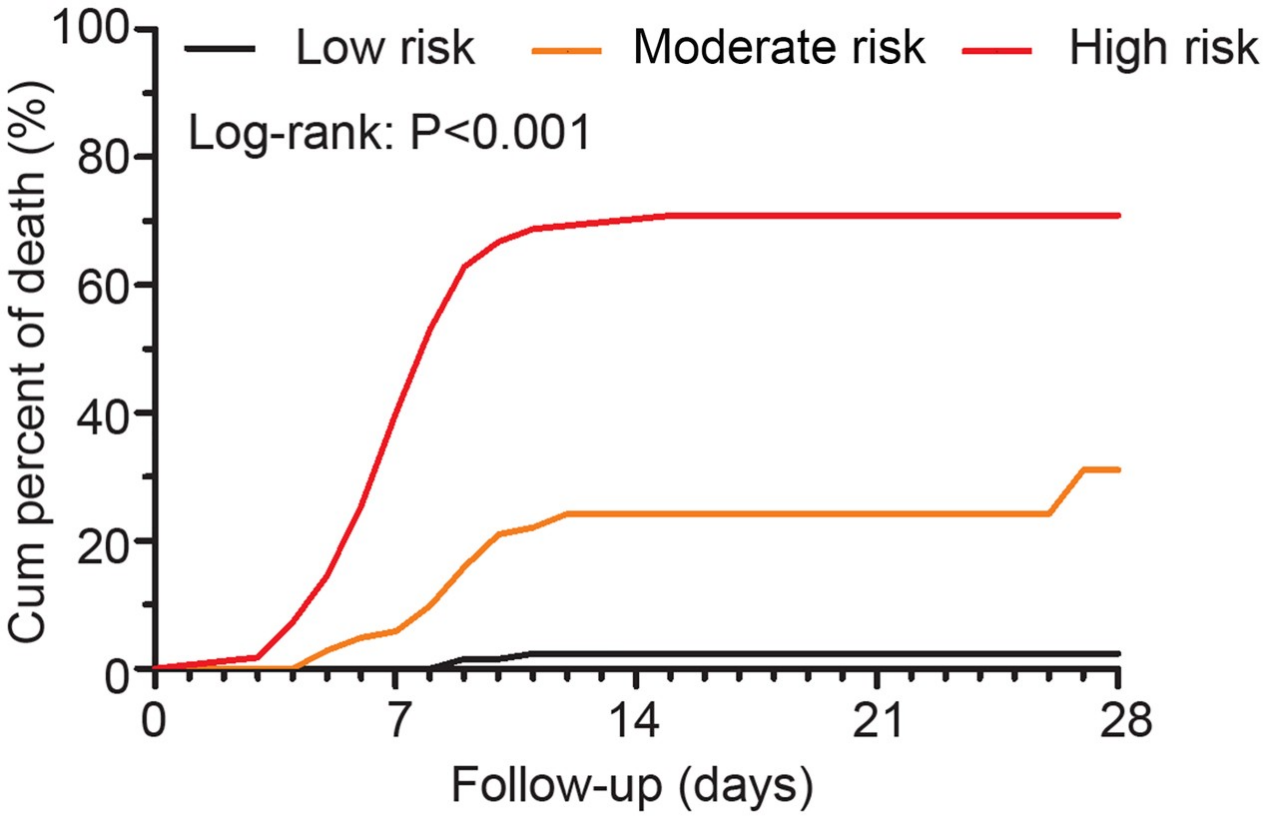
Kaplan-Meier curves of risk stratification for cumulative mortality in the primary cohort of SFTS according to the prognostic scores from the nomogram.

According to the nomogram we established, patients were divided into three levels of death risk: low, moderate, and high. As shown in Table 5, among the patients with <18 points, only six patients died (2.2%). There were 25 patients with 18–26 points who died (24.0%, HR = 11.957). If patients scored points ≥27, the mortality rate was 69.1% (HR = 57.768). The Kaplan-Meier curves showed cumulative mortality in patients with different risk levels in primary cohort. The cumulative mortality was significantly different among the three groups (Fig 4).

**Table 5:** Death risk prognosis group according to nomogram score.

Group	Score	All	Non-fatal		Fatal		HR (95%CI)
			N	%	N	%	
Low	~18	270	264	97.8	6	2.2	1.000
Moderate	~27	104	79	76.0	25	24.0	11.957 (4.903~29.163)
High	~40	55	17	30.9	38	69.1	57.768 (24.317~137.234)
All	-	429	360	83.9	69	16.1	-

## Discussion

SFTS is widely distributed in China, being endemic in more than 20 provinces [7,8]. The mortality of SFTS ranges from 11.2% to 30%. In addition to China, SFTS has been reported in Korea, Japan, and the United States [16,24]. The incidence, clinical manifestations, and mortality of SFTS are similar to those in China, especially in South Korea. There are outbreaks by nosocomial infection, and the mortality is much higher than the rate reported in China [5,6]. This study aimed to analyze the clinical information of patients with early-stage SFTS and to establish a nomogram for the mortality risk. The resulting nomogram is a simple and practical clinical scoring system, through which we can identify critically ill patients and provide intensive medical intervention for patients as soon as possible to reduce mortality.

In the present study, 69 patients died (16.1%). Sex was not associated with mortality, but the mean age was significantly higher among those who died, as supported by previous studies [11,25,26]. Older patients usually have a higher incidence and duration of comorbidities and poor compensatory organ function, which might explain, at least in part, the higher mortality than that observed in younger patients. The mortality rate of SFTS is high, but the CDC reports contrary results [27]. The possible reasons for this discrepancy may include: 1) frontline physicians may have insufficient understanding of this disease and fail to diagnose it; and 2) many hospitals are unable to detect SFTSV and cannot make the accurate diagnosis in the first place. Therefore, there is an urgent need to enhance the diagnosis and management of this disease through a comprehensive reporting system, education about SFTS, exploration of the risk factors for death, early identification of critically ill patients, and early intensive medical intervention.

In addition to systemic symptoms, patients who eventually die show higher frequencies of vomiting, diarrhea, petechia or ecchymosis, and lymphadenopathy [28–30], but those symptoms and signs were not observed in the present study. On the other hand, we observed that the frequency of encephalopathy in the mortality group was higher than in the survival group, while the frequency of headache was higher in the survival group. The patients in the mortality group might report headache less frequently due to disturbance of consciousness at admission and inability to accurately describe their complaints. Several previous studies reported that manifestations of the central nervous symptom (CNS) such as apathy, hypersomnia, coma, muscle twitching, and hyperspasmia were high-risk factors of death in patients with SFTS [31–33]. Park et al. [34] reported that the CNS manifestations of SFTS, including acute encephalopathy/encephalitis, are common complications of SFTS. Although meningeal inflammation is infrequent in patients with SFTS-associated encephalopathy/encephalitis (SFTSAE), SFTSV is frequently detected in the cerebrospinal fluid (CSF), along with elevated monocyte chemoattractant protein 1 (MCP-1) and IL-8. These findings indicate that direct CNS invasion by the SFTSV with elevated cytokine levels in the CSF may play an important role in the pathogenesis of SFTSAE [34]. In addition, Masahiko et al. performed an autopsy of a Japanese patient who died of SFTS and found that the rapidly progressive CNS involvement in patient with SFTS might be a result of direct damage caused by infiltration of SFTSV-positive cells into the brain tissues [35]. Therefore, CNS damage, exhibited as encephalopathy in early stage, is a strong predictor of critical outcomes.

In the present study, laboratory parameters showed that the levels of platelets and leukocytes were reduced in patients who died, while neutral granulocytopenia was predominant and hemoglobin was normal. On the other hand, the nadir of white blood cells and thrombocytopenia were not significantly related to fatal outcomes [36,37]. While the level of leukocytes and lymphocytes are both decreased in both groups, their percentages in fatal patients are significantly lower than survivors. These results suggest that the damage or consumption of lymphocytes is more serious in non-surviving patients, indicating more severe acquired immune injury in non-surviving patients. Li et al. [38] reported that decreased percentage and number of CD4 T cells, including Th1, Th2, and regulatory T cells (Tregs), were associated with an increased severity of SFTS. In addition, increased percentages of Th2, Th17 and Tregs in residual CD4 T cells, as well as skewing of Th17/Treg and Th2/Th1 ratios, may cause poor outcomes in SFTS patients. Hu et al. found that inflammatory mediators remained at high levels when death occurred, while they recovered within 3 weeks in surviving patients, indicating that the occurrence of immunologic injury and inflammatory storm in SFTS patients were associated with the severity of SFTS [39]. The mortality of SFTS might be related to immunologic injuries [39,40].

In addition to the hematologic system, SFTS can also cause multiple organ damage such as to the myocardium, coagulation function, liver, and kidney. Consistent with previous research [36,37], we also observed that parameters of myocardial injury such as LDH, CK, CK-MB, and AST levels were elevated in most patients with SFTS who do not survive. The higher the LDH levels, the higher the mortality of SFTS. This result is in accordance with the conclusion of reports by Gai et al. [41] and Cui et al. [42]. In the present study, some patients with fatal outcomes exhibited arrhythmia, pulmonary edema, and circulatory failure, which might be the actual cause of death. While the specificity of LDH is limited, its sensitivity is high; therefore, when it increases shortly after infection, along with elevated CK, CK-MB, and AST levels, and especially when cardiac dysrhythmia or dysfunction occurs, it could be a sensitive predictor of fatal outcomes for patients with SFTS.

Liver damage is a significant manifestation of SFTS [28,36,42]. Most of the patients with SFTS had abnormally elevated ALT and AST, but those levels were further higher in the mortality group. We also found that APTT prolongation is common in SFTS patients, especially in the mortality group, with a significantly longer APTT than in survivors. On the other hand, there was no significant jaundice. Furthermore, the ratio of AST/ALT was much higher, suggesting that the AST levels not only indicate acute liver damage, but also reflect the presence of myocardial injury. These results indicate that although patients with SFTS have severe liver inflammation and coagulation abnormalities in the early stage of the disease, liver failure do not occur in all patients who die. While SFTS infection affects the liver function, it is probably not the main cause of death.

sCr and BUN levels were mildly elevated in the mortality group. The multivariable analysis showed that BUN was an independent risk factor for fatal outcome, as supported by previous studies [11,43]. The main causes of increased BUN could be high protein diet, decrease in glomerular filtration rate (GFR) and blood volume (hypovolemia), congestive heart failure, gastrointestinal hemorrhage, fever, and increased catabolism. Elevated BUN plays a role in predicting fatal outcome, indicating that BUN reflects the general disease conditions after the onset of SFTS, which include fever, poor nutritional status, increased catabolism, impaired early renal function, and/or insufficient circulating blood volume.

Based on the univariable and multivariable Cox regression analyses, we established for the first time a mortality risk predictive nomogram for SFTS. Five independent risk factors (older age, early-stage encephalopathy, decreased lymphocyte percentage, and elevated serum concentration of BUN and LDH) were found to be associated with 7-, 14-, and 28 days mortality. The C-index of the prognostic nomogram is 0.91, which shows high value for predicting fatal outcome. The nomogram was applied to the original cohort and the calibration plots showed fair concordance between the nomogram predictions and actual observations for 7-, 14-, and 28-days overall survival. These results strongly support the usefulness of the nomogram in patients with SFTS. Moreover, according to the scoring system, the patients can be divided as low-, moderate-, and high-risk mortality. previous studies attempted scores for predicting the outcomes of SFTF. Wang et al. [25] established a scoring system using 174 patients and in which the AUC was 0.894. In the system by Xiong et al. [31], the viral load is included, but not all hospitals in China are able to determine the exact viral load and this scoring system is impractical. In addition, their score relies on a complicated formula. Jia et al. [43] developed a simple scoring system based on three variables, but it also has a complicated formula. The prominent advantages of our nomogram include that all factors can be obtained through routine examinations, in a timely manner soon after admission, and the score sheet is concise. The nomogram provides fast graphical calculations of complicated formulas to a practical precision. It is practical and intuitional for primary care physicians in the countryside, who are likely to be the first-line doctors to identify this infectious disease. In addition, similar nomograms are available for other infectious diseases such as acute-on-chronic liver failure, bacteremia-urinary tract infections (b-UTI), maternal cytomegalovirus serostatus, and risk of HIV acquisition [44–47].

At present, the pathogenesis of SFTS and the exact cause of death are still unclear and there is no specific antiviral treatment available, which is the reason why the mortality rate is still so high. By identifying risk factors for death of SFTS, our work provides useful information for the study of the pathogenesis of this viral infection. We found that age, percentage of lymphocytes, and BUN were host factors. Elevated LDH level and early-stage encephalopathy indicate that the severe injuries to the myocardium and central nervous system by the viral infection are major causes of increased risk of death. In addition, *in vitro* and *in vivo* studies showed that favipiravir was able to inhibit SFTSV virus replication [48,49]. We are conducting a clinical trial of favipiravir treatment of SFTS. Classification of the patients according to the severity of the disease at the early stage using the scoring system we established in this study will benefit further research on drug efficacy and side effects.

Our study has some limitations. First, some patients have given up on further medical treatment because of financial conditions when the disease progressed, and some severely ill patients were lost to follow-up. Second, due to the limited laboratory resources in some primary hospitals, viral load detection of SFTSV could not be conducted at all centers. Third, post-mortem examination was not performed in all cases. Finally, the nomogram we established needs to be improved by external prospective cohorts. For this reason, we are undertaking a prospective study on a larger cohort of SFTS patients hospitalized since September 2018 to validate the predictive value of this model.

In conclusion, SFTS is broadly distributed in central and northeastern China. The mortality rate is high. The main clinical manifestations are acute fever, thrombocytopenia, and leukopenia, often accompanied multiple organ damage, such as CNS, myocardial, liver, and coagulation function. The severe injuries of the myocardium and central nervous system caused by SFTSV infection are major causes of increased risk of death. Older age, early-stage encephalopathy, decreased lymphocyte percentage, and elevated serum levels of BUN and LDH are independent predictors of death. We established a simple and practical clinical scoring system, through which the patients can be classified as low-, moderate-, and high-risk of mortality. We can identify critically ill patients and provide intensive medical intervention for patients as soon as possible, which may contribute to reducing the mortality of SFTS.

## Supporting information

S1 Fig Distribution map of the five study centers. (TIF)

S1 Checklist STROBE checklist. (DOC)
