# Supplementary figures and images for: A nomogram to predict mortality in patients with severe fever with thrombocytopenia syndrome at the early stage—A multicenter study in China
Source: PLoS Negl Trop Dis. 2019 Nov 25;13(11):e0007829. doi: 10.1371/journal.pntd.0007829 (PMC6934327; doi:10.1371/journal.pntd.0007829)

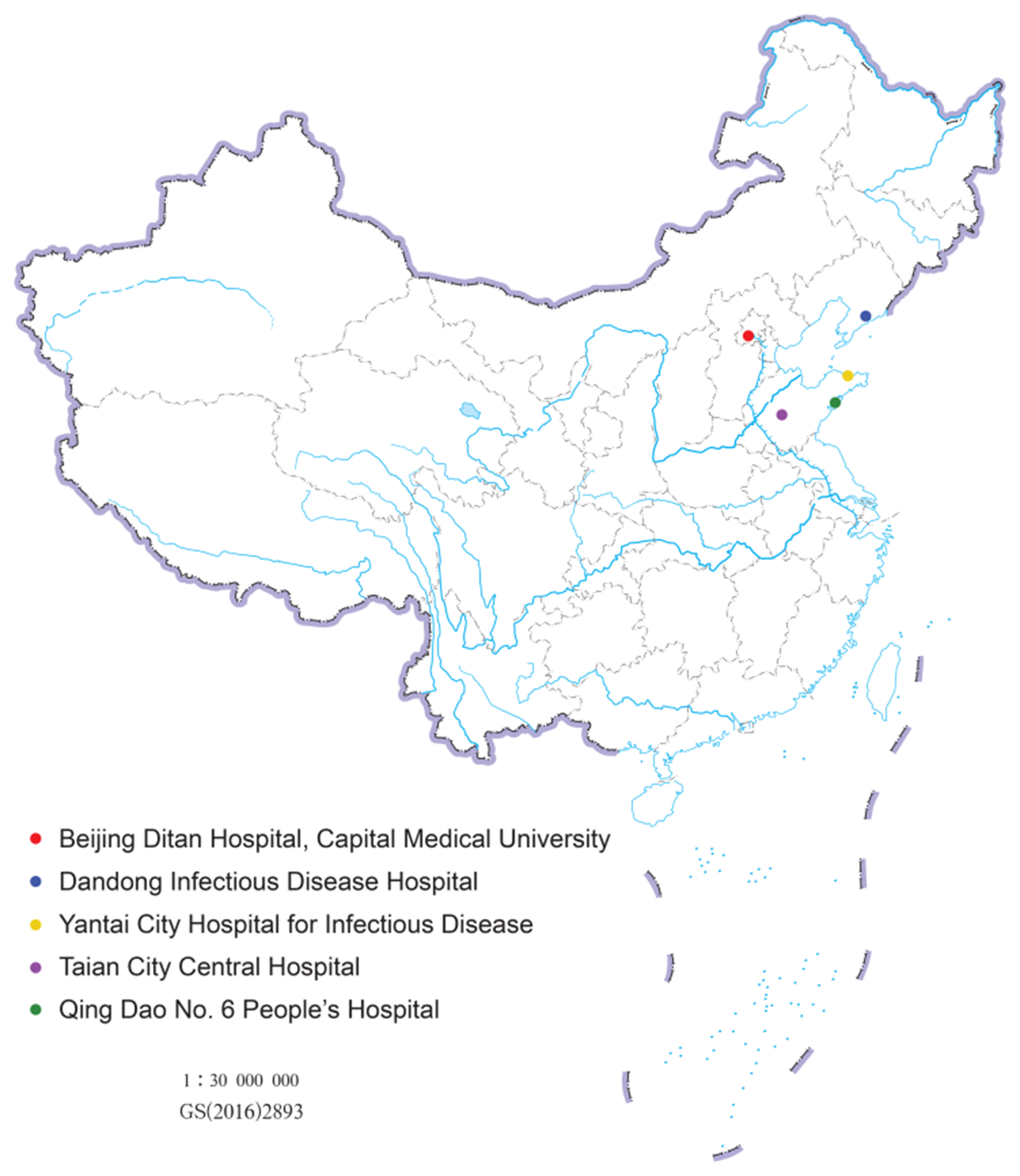

Supplement: S1 Fig — (TIF) [file pntd.0007829.s001.tif]
